# The Impact of Next-Generation Sequencing Workflows on Outcomes in Advanced Lung Cancer: A Retrospective Analysis at One Academic Health System

**DOI:** 10.3390/cancers16213654

**Published:** 2024-10-30

**Authors:** Chetan V. Vakkalagadda, Danielle B. Dressler, Zequn Sun, Joseph Fuchs, Yingzhe Liu, Philip Silberman, Avanthi Ragam, Sheetal Kircher, Jyoti D. Patel, Nisha A. Mohindra

**Affiliations:** 1 Division of Hematology and Oncology, Department of Medicine, Feinberg School of Medicine, Northwestern University, Chicago, IL 60611, USAnisha.mohindra@nm.org (N.A.M.); 2 Knight Cancer Institute, Oregon Health and Science University, Portland, OR 97239, USA; 3 Division of Hematology Oncology, Vanderbilt University Medical Center, Nashville, TN 37235, USA; 4 Enterprise Data Warehouse, Clinical and Translational Sciences Institute, Feinberg School of Medicine, Northwestern University, Chicago, IL 60611, USA; 5 Northwestern Medicine Delnor Hospital, Geneva, IL 60134, USA

**Keywords:** biomarkers, lung cancer, reflex testing

## Abstract

Purpose: Broad-based molecular testing with next-generation sequencing (NGS) is now the standard of care in advanced non-small cell lung cancer (NSCLC). Two approaches to molecular testing are (1) reflexive testing at pathologic NSCLC confirmation, often using an in-house molecular panel, and (2) send-out testing to private vendors, ordered by a clinician. This study explored the outcomes with reflex versus send-out testing. Methods: A retrospective chart review was conducted of patients diagnosed with de novo stage IV NSCLC in 2019 and 2020 at three hospitals in the same system, one academic hospital (Northwestern Memorial Hospital, or NMH) utilizing reflex, in-house NGS, and two community-based hospitals (Central DuPage Hospital, or CDH, and Delnor, or D) sending out tissue samples for testing. The outcomes assessed were the time from biopsy to results, biopsy to treatment, the incidence of first-line targetable mutations and the use of first-line targeted therapies, and overall survival. Results: In total, 191 patients met the inclusion criteria, 85 at NMH, 106 at CDH + D, and in total, 131 in 2019 and 60 in 2020. The time to results was significantly shorter with reflexive NGS when compared with send-out testing; the time to treatment was also shorter but not statistically significant. At CDH + D, the time to results was significantly shorter with a limited panel than with comprehensive testing, but the time to treatment was similar. NGS testing rates were 95% at NMH and 84.5% at CDH + D (*p* = 0.009), with 31.0% at NMH receiving 1L targeted therapies versus 20.8% at CDH + D (*p* = 0.08). In 2019, the median time from biopsy to treatment was 35 days at NMH and 38 days at CDH and Delnor; in 2020, time to treatment was 26 days and 37 days, respectively. Overall survival trended longer in 2020 relative to 2019 independent of site. Conclusion: Reflexive NGS testing is associated with a shorter time to actionable results and higher rates of first-line targetable mutations than send-out testing. In practices with send-out testing, limited panels had slightly faster turnaround times but no difference in time to treatment. If resources allow, reflexive NGS should be considered in healthcare systems for patients with NSCLC.

## Simple Summary

Molecular testing is becoming the standard of care across multiple tumor types in oncology. In non-small cell lung cancer (NSCLC), the uptake of molecular testing with next-generation sequencing (NGS) has been earlier than in other fields due to the clinical relevance of the molecular results. There are multiple approaches to NGS, ranging from sending testing internally, often reflexively, to referring externally to limited or comprehensive panels. This study aims to identify the differences in time to results, time to treatment, molecular alterations, and clinical outcomes for different molecular workflows in NSCLC. The results may impact the workflows for testing in a variety of cancers.

## 1. Introduction

Lung cancer has historically accounted for the most cancer-related deaths in the United States (US), and approximately 84% of lung cancers are non-small cell lung cancer (NSCLC) [1,2]. Most patients who present with NSCLC are diagnosed at advanced stages (stage III or IV) [2]. In the past decade, the identification of driver mutations as a key factor in NSCLC oncogenesis has changed the landscape of NSCLC therapy. In the metastatic setting, the United States Food and Drug Administration (FDA) has approved targeted therapies against classical and atypical EGFR mutations, ALK fusions, ROS1 alterations, BRAF V600E mutations, NTRK fusions, RET fusions, MET alterations, Her2 mutations, and KRAS mutations, and these are reflected in the guidelines of the National Comprehensive Cancer Network (NCCN) [3]. Such therapies have improved the outcomes for patients with advanced disease compared to the standard of care for patients without targetable alterations [4,5]. Targeted therapies are also now FDA-approved in the adjuvant setting for EGFR-mutated cancers and ALK-mutated cancers per the ADAURA, LAURA, and ALINA trials [6,7,8]. Accordingly, the NCCN guidelines for NSCLC recommend a broad, panel-based approach for biomarker testing [3].

Such driver alterations are identified by molecular testing, often with next-generation sequencing (NGS). The uptake of NGS has been heterogeneous across practice settings. Understanding the mechanics of biomarker testing is important as having any biomarker testing or results-concordant therapy available is associated with improved treatment and survival outcomes [9,10]. Timely molecular results are likewise important as some patients require prompt initiation of therapy, yet choosing inaccurately matched therapy can adversely impact the outcome—for instance, upfront checkpoint inhibitors in patients with EGFR or ALK mutations are ineffective and even harmful [11,12,13,14]. While the uptake of molecular testing has increased over time, and upfront circulating tumor DNA NGS testing can improve time to treatment in NSCLC, features of healthcare systems or systemic bias can adversely impact the frequency and breadth of NGS testing [15,16,17,18,19]. 

**Figure 1:**
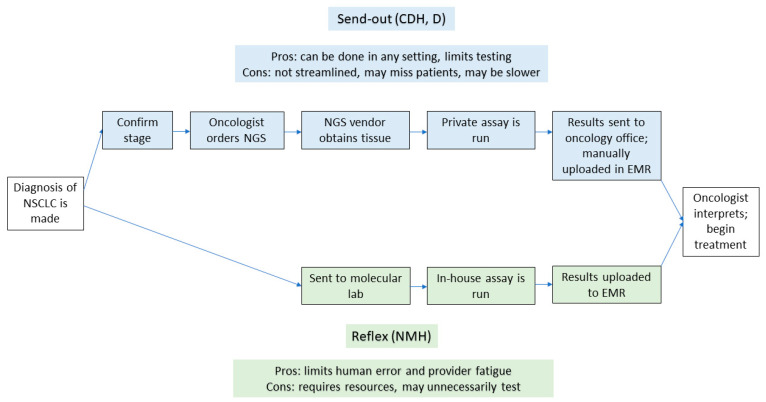
Flowchart with the steps in both send-out and reflexive testing. The upper part of the figure denotes the steps involved with send-out testing, while the lower part shows the steps in reflexive testing. EMR—electronic medical record; NMH—Northwestern Memorial Hospital; CDH—Central DuPage Hospital; D—Delnor Hospital.

There remain ongoing challenges to optimizing the workflows for NGS testing. Further, workflows for NGS testing differ for send-out testing compared to reflexive testing. Historically, NGS had been sent out to private vendors by a patient-facing provider once they were made aware of a diagnosis of NSCLC (send-out testing). As platforms for NGS have scaled upward, and as more institutions are able to offer in-house testing, some systems have transitioned to a reflexive approach [20]. In this model, once a pathologist sees a diagnosis of NSCLC, a sample is sent to a molecular lab for testing, bypassing clinician input for ordering a test (reflexive testing). The schematics of workflows in each scenario, and the associated advantages and disadvantages of each approach, can be seen in Figure 1. 

System-wide factors such as available resources, reimbursement models, or state laws are likely to encourage the use of one approach over the other [21]. However, a comparison of one approach to the other is worthwhile given the increased need for molecular testing. To the authors’ knowledge, a comparison of reflexive versus send-out NGS has not yet been conducted, nor has an analysis been carried out using patient-level data. This comparison across multiple hospitals in the same system was the purpose of this study.

## 2. Methods

### 2.1. Study Design

This study was designed to determine the timeframe of NGS testing in advanced NSCLC. The primary objective was to compare the difference between the time from biopsy to both NGS results and the initiation of systemic treatment with reflexive NGS compared to a setting with send-out testing. Secondary objectives included the following:

- When send-out testing was conducted, to carry out a comparison of time from biopsy to results and biopsy to treatment between patients with comprehensive send-out testing and select send-out panels.
- Whether reflexive NGS was associated with differences in mutational status, treatment allocations, and survival compared with send-out testing at CDH + D.

### 2.2. Setting and Subjects

Northwestern Medicine is a network of 13 hospitals across the Chicago metropolitan area in Northern Illinois, USA, across which NGS testing methods vary. This study focused on 3 hospitals: Northwestern Memorial Hospital (NMH), the 891-bed flagship academic referral center in downtown Chicago; Central DuPage Hospital (CDH), an affiliated hospital in the western suburbs of Chicago; and Delnor Hospital (D), an affiliated hospital in the western suburbs of Chicago. At NMH, upon histologic confirmation of NSCLC, the tissue is sent by pathology to the molecular lab for further testing with an in-house panel of over 500 genes and fusions. This panel is sent independent of squamous or non-squamous histology and independent of stage. At CDH and Delnor, NGS testing workflows vary by provider: there is no set standard for testing, and tissue is sent by oncologists to the private vendor of their choice and, therefore, can also depend upon stage and histology. 

For analysis purposes, CDH + D were combined into one cohort, as both are affiliated with Northwestern and located in the western suburbs with similar practice patterns and NGS testing. 

This investigation was limited to 2019 and 2020 to capture the impact of the COVID-19 pandemic on time to treatment. It has been well established that the COVID-19 pandemic led to delays in cancer diagnoses and preventable deaths from theoretically non-fatal illnesses [22].

### 2.3. Data Collection

This was a retrospective chart review, conducted by physicians on the research team (CVV, DBD, JF), of all patients with a new diagnosis of stage IV NSCLC between January 1, 2019, and December 31, 2020, at Northwestern Memorial Hospital (NMH), Central DuPage Hospital (CDH), and Delnor Hospital (D). Patient records were obtained through the Northwestern Medicine Enterprise Data Warehouse (EDW) with a formal request through the METRIQ OncoSET precision medicine database. Medical records were then reviewed in the electronic medical record (EMR). 

The inclusion criteria for analysis were a new diagnosis of stage IV non-small cell lung cancer; diagnostic evaluation conducted at NMH, CDH, or D; and being age 18 years or older at time of diagnosis. 

After data were obtained from the Enterprise Data Warehouse, physician members of the research team reviewed patient charts to obtain the following:

- Patient data, including smoking status, stage of disease, PDL1 status, NGS results on tissue, NGS results from blood, and whether the patient was enrolled on a clinical trial.
- Clinical information, including pathologic diagnosis, first-line systemic treatment chosen for each patient, date of initiation of therapy (defined as the first date that the patient received therapy), date of disease progression, specific therapies chosen beyond first-line treatment, and date of death or last follow-up.
- The system’s information, including hospital of diagnosis and treatment, date of biopsy for new diagnosis of lung cancer, date of ordering NGS, date of NGS results, whether “insufficient tissue” was identified on biopsy and a repeat biopsy was needed, and the method of NGS sequencing (whether in-house or sent out).
- Among the biopsies sent out (CDH + D), the team reviewed whether NGS was comprehensive or limited. Limited panels were defined as those requiring a clinician to select certain oncogenic drivers, while comprehensive included sending for a panel designated by the vendor.

### 2.4. Power Analysis

A total of 191 patients met the inclusion criteria, with 85 patients at NMH and 106 patients at CDH + D. Group sample sizes of 85 in the NMH group and 106 in the CDH + D group achieved 80% power to detect a difference of 11.6% in the NGS testing rate based on the one-sided Fisher’s Exact Test, with a significance level of 0.05. This post hoc power analysis demonstrated that the study was sufficiently powered, consistent with the observed rates of 95% at NMH and 84.5% at CDH + D.

### 2.5. Data Analysis

The statistical analyses encompassed descriptive summaries, comparative assessments, survival analyses, and multivariate modeling to investigate the impact of NGS testing methods on the outcomes in advanced NSCLC patients. Descriptive statistics were employed to summarize demographics and clinical characteristics, while comparative analyses utilized chi-square tests to compare proportions of NGS testing and assess differences in first-line targeted therapies and other categorical variables between NMH and CDH + D. The time from biopsy to treatment initiation was compared using the Wilcoxon rank sum test. The survival analyses conducted via Kaplan–Meier curves and log-rank tests explored overall survival across various cohorts. Cox proportional hazards regression models were employed for multivariate analyses, adjusting for potential confounding variables, with the proportional hazards assumption validated using Schoenfeld residuals. Subgroup and sensitivity analyses were conducted to explore the specific characteristics of NGS testing. The statistical approach aimed to provide an assessment of the research questions while acknowledging the limitations inherent in retrospective analyses and healthcare system variations.

### 2.6. Limitations

Potential confounding factors and biases inherent in retrospective studies, as well as variations in healthcare systems, were acknowledged as limitations. These were addressed through statistical adjustments and sensitivity analyses where applicable.

## 3. Results 

### 3.1. Demographics

The initial query yielded a total of 864 patients. After chart review, 191 patients with stage IV disease diagnosed at NMH, CDH, or Delnor met the inclusion criteria. In total, 68.6% (131/191) were diagnosed in 2019 and 31.4% (60/191) were diagnosed in 2020. The demographics are shown in Table 1. 

### 3.2. Time to Treatment

The median time from biopsy to actionable NGS results was 19 days at NMH and 24 days at CDH + D (*p* < 0.001) (Table 2). The median time from first biopsy to treatment was 30 days at Northwestern and 37 days at CDH + Delnor (*p* = 0.092). Both are shown in Table 2. 

**Figure 2:**
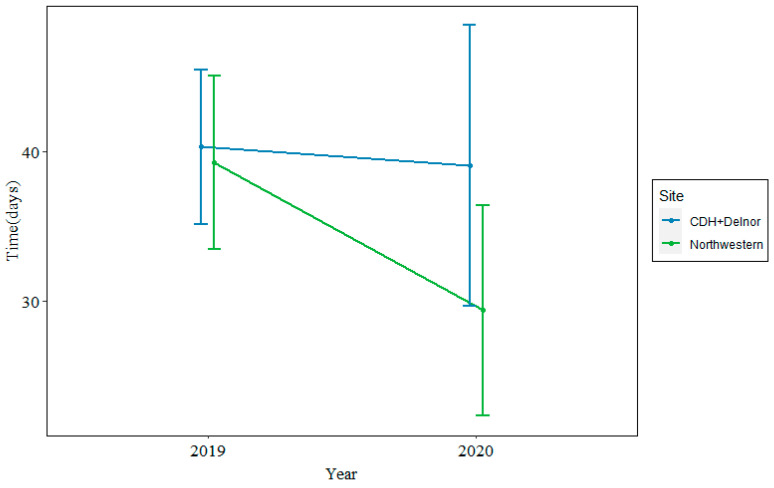
Median time from biopsy to treatment at Northwestern (green) vs. CDH + Delnor (blue) in 2019 (left) and 2020 (right) in days.

In 2019, the median time from biopsy to treatment was 35 days at Northwestern and 38 days at CDH + Delnor. In 2020, during the COVID-19 pandemic, the median time from biopsy to treatment was 26 days at Northwestern and 37 days at CDH + Delnor. This is shown in Figure 2.

At CDH + D, the time from biopsy to actionable NGS results was 26 days with comprehensive testing and 22 days with non-comprehensive testing (*p* = 0.033). The time to treatment was 37 days with comprehensive testing and 38 days with non-comprehensive testing (*p* = 0.6). This is shown in Table 3. 

### 3.3. NGS Testing Outcomes

**Figure 3:**
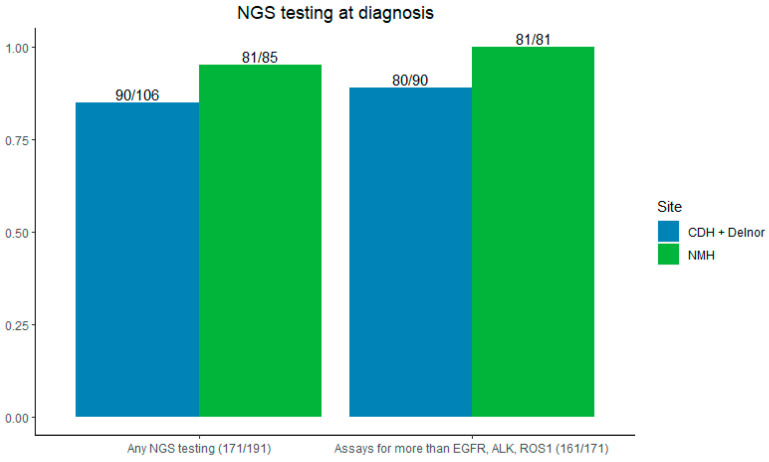
**Left**: rates of NGS testing by site. **Right**: NGS testing for more than EGFR/ALK/ROS1. CDH + Delnor in blue and NMH in green.

The majority of patients (89.5%) had NGS testing sent at the time of initial biopsy—95% (81/85) at NMH and 84.5% (90/106) at CDH and Delnor (*p* = 0.009). Most NGS testing evaluated for more than EGFR, ALK, and ROS1 (94.2%), 100% at NMH, and 88.9% (80/90) at CDH + Delnor (Figure 3). 

At Northwestern, 29.6% (24/81) of patients had targetable mutations for first-line therapy; at CDH + Delnor, 21.1% (19/90) of patients had such mutations (*p* = 0.10).

**Figure 4:**
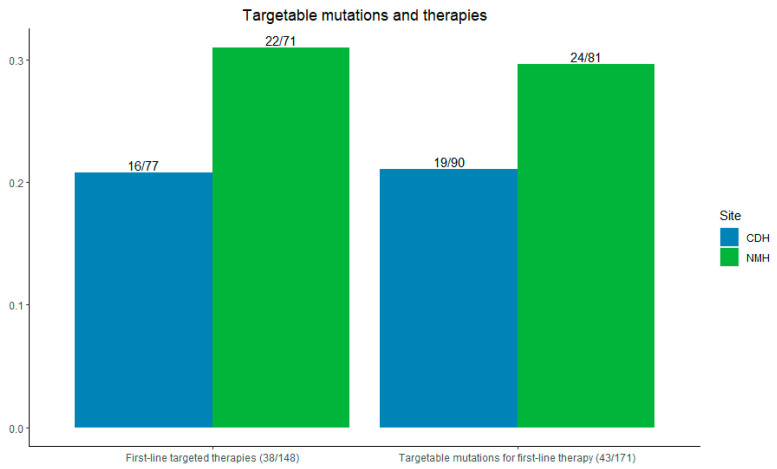
**Left**: incidence of first-line targetable therapies initiated. **Right**: incidence of first-line targetable mutations seen by site. CDH + Delnor in blue and NMH in green.

Among all patients, 77.4% (148/191) received any systemic therapy—83.5% (71/85) at NMH and 72.6% (77/106) at CDH + Delnor. First-line targeted therapies were used in 31.0% of patients at Northwestern (22/71) compared with 20.8% at CDH and Delnor (16/77) (*p* = 0.08), as shown in Figure 4. 

Mutations seen at each hospital are listed in Supplemental Table S1. At NMH (reflexive), among the 24 patients with targetable mutations, 18 had an EGFR, ALK, or ROS mutation, and 6 had other mutations (4 MET, 1 BRAF V600E, or 1 NTRK3). At CDH + D, 17 had EGFR/ALK/ROS mutations and 2 had other mutations (1 MET and 1 BRAF V600E).

More patients were diagnosed in 2019, 68.6% (131/191), than 2020, 31.4% (60/191). Among the patients who received systemic therapy (n = 148), 102 were diagnosed in 2019 (44 at NMH and 58 at CDH and Delnor) and 46 in 2020 (27 at NMH and 19 at CDH and Delnor). Prior to starting systemic therapy, 59/148 patients required radiation (29 NMH, 30 CDH + Delnor) and 20/148 required a repeat biopsy (10 NMH, 10 CDH + Delnor). 

### 3.4. Overall Survival

Figures S1a, S1b, and S1c show the median survival overall, by year, and by site. The median overall survival was 13 months. By year, the median survival was 11.03 months in 2019 and 17.43 months in 2020 (*p* = 0.2). By site, the median survival was 14.9 months at Northwestern and 10.78 months at CDH + Delnor (*p* = 0.11). 

## 4. Discussion

This analysis of NGS testing outcomes in advanced NSCLC at three separate hospitals in the same healthcare system—one academic hospital and two affiliate hospitals—shows that reflexive NGS testing is associated with more rapid turnaround times and a trend towards a shorter time to treatment. Comprehensive send-out testing was associated with slightly longer turnaround times relative to limited send-out testing, but there was no statistically significant difference in the time to the initiation of treatment. 

Our assessment of NGS testing across our network revealed other valuable insights. Nearly 90% of patients overall had NGS testing sent at the time of initial biopsy, with significantly higher rates when carried out reflexively. The vast majority had testing for, at minimum, EGFR, ALK, and ROS1, which was the standard of care in 2019 and 2020 [23]. This rate is comparable and even higher than that reported in the literature when evaluating NGS testing across multiple centers or within one hospital system [24,25]. It is important to note that our review of NGS testing occurred between 2019 and 2020, compared to previous studies which occurred between 2017 and 2019, which may reflect the advances and availability of NGS testing across time and the recommendation for testing within treatment guidelines [26]. However, this shows a trend toward improved rates of NGS testing in accordance with NCCN guidelines for the treatment of NSCLC [3].

The time to actionable results was shorter at NMH compared with CDH + Delnor, with a trend toward a shorter time to treatment when looking at both years of data. The time to treatment was similar between hospitals in 2019, but markedly shorter at NMH in 2020 than at CDH + D in 2020. This raises the question of whether internal testing bypassed any supply chain disruptions that would have impacted send-out testing during the COVID-19 pandemic, as we know that supply chains for healthcare, and oncology specifically, were disrupted in that period [27,28]. The analysis of the time to treatment at CDH + Delnor alone noted that whilst comprehensive send-out testing had slightly longer turnaround times when compared with limited panels, there was no quantitative difference in the time to the initiation of treatment.

A higher percentage of patients at NMH had targetable alterations and were treated with first-line targeted therapies when compared with CDH + D, but this finding was not statistically significant given the lack of power in small sample sizes. One explanation could be that a limited panel, only carried out with send-out testing, could have missed mutations beyond EGFR/ALK/ROS1. The specific mutations per Supplementary Table S1 suggest that despite similar numbers of EGFR/ALK/ROS1 between the reflexive and send-out groups (18 and 17, respectively), the reflexive group had six patients with mutations beyond EGFR/ALK/ROS1 while the send-out had two. A higher percentage of patients at NMH compared to CDH + D were never or former smokers (85.9% vs 76.4%), which could also correlate with actionable genomic alterations and first-line targeted therapies. However, due to the lack of statistical significance, we cannot conclude that reflexive NGS testing is associated with higher rates of identifying alterations when compared with send-out testing based on these data alone. 

Overall survival trended longer in 2020 than 2019 at both sites, while prior data showed a decreased time to treatment in 2020 only at Northwestern. This suggests that factors apart from NGS testing are responsible for improved overall survival. At least five targeted therapies were either newly approved or had expanded approvals designated in 2020 for non-small cell lung cancer [29]. While final survival data for some of these agents are still pending, we suspect these approvals had a greater impact on overall survival than patterns of NGS testing. It is also likely that the COVID-19 pandemic led to a survival bias in 2020; patients who may have sought care in 2019 may have chosen to stay home in 2020 rather than engaging with the healthcare system and risking COVID-19 infection. We suspect that this bias explains the large difference in patients between 2019 and 2020—131 and 60, respectively. 

There are several limitations to this study. As mentioned above, confounders and limitations inherent in retrospective studies could have impacted the results, but they were adjusted for using sensitivity analyses where possible. Contextual factors beyond NGS testing procedures likely contributed to differences in study outcomes but could not be evaluated in this retrospective study design, such as clinician characteristics, hospital resources, and clinic staffing. We combined CDH and Delnor given the similarity of catchment area and to maintain relatively equal sample sizes across cohorts, but there could have been other variability between the two sites related to patient characteristics, insurance coverage, and NGS testing vendors. With NMH being a referral center, it is possible that the patient populations differed significantly between NMH and CDH + Delnor. We aimed to limit this bias by only including patients with a pathologic diagnosis of NSCLC at each site. We also explored whether rates of radiation therapy or repeat biopsy differed between cohorts as these could impact the time to treatment and survival. Rates of each were similar between NMH and CDH + D, removing this as a potential confounding factor.

There remain several ongoing questions related to molecular testing. In addition to cost evaluations, factors such as patient preferences related to treatment delays and clinician burnout should be explored. There was no consideration of cost or insurance in this analysis, which, in clinical practice, would influence the choice of specific NGS assays over others. A cost–benefit analysis could be considered when looking at the costs of in-house, reflexive testing compared with select testing, particularly when related to insurance reimbursement for patients with an earlier-stage disease who would not immediately benefit from targeted therapies. A second consideration relates to physician burnout. Clerical tasks such as computerized physician order entry (CPOE) have been shown to be associated with higher risk of burnout in a large survey of United States physicians [30]. Removing this burden from clinicians and building it into the healthcare system may improve clinician well-being and could merit further study; this benefit would also not be captured in a conventional cost–benefit analysis.

With the median overall survival ranging between 10 and 15 months in our analysis, turnaround times of 30 to 37 days represent a notable portion of a patient’s life after the life-altering diagnosis of advanced NSCLC. Working to shorten this duration and the resultant anxiety about next steps for patients and their families is a worthwhile goal, independent of the objective benefits demonstrated in this work. As the healthcare system recovers from the COVID-19 pandemic and further challenges from its wake continue to emerge, reflecting on practice patterns will be essential to ensuring that systems can be more resilient if a similar disruption occurs again. 

## 5. Conclusions

Reflexive NGS testing was associated with a shorter time to actionable results. Hospital systems will need to evaluate whether they have the capacity for reflexive in-house testing for patients with advanced NSCLC. Additionally, clinicians and healthcare systems may need to assess when limited panels versus more comprehensive panels should be considered.

## Figures and Tables

**Table 1:** Demographics.

Variables	n = 191 ^1^	NMH, n = 85 ^1^	CDH + Delnor, n = 106 ^1^
Age	70 (62, 78)	68 (61, 79)	71 (63, 78)
Missing	3	2	1
Gender			
Female	107 (56.0%)	51 (60.0%)	56 (52.8%)
Male	84 (44.0%)	34 (40.0%)	50 (47.2%)
Race			
Asian	12 (6.3%)	8 (9.4%)	4 (3.8%)
Black or African American	22 (11.5%)	18 (21.2%)	4 (3.8%)
Other	14 (7.3%)	8 (9.4%)	6 (5.7%)
White	143 (74.9%)	51 (60.0%)	92 (86.8%)
Smoking Status			
Never	41 (21.5%)	19 (22.4%)	22 (20.8%)
Former	113 (59.2%)	54 (63.5%)	59 (55.7%)
Current	37 (19.4%)	12 (14.1%)	25 (23.6%)
Site			
CDH	86 (45.0%)		
Delnor	20 (10.5%)		
NMH	85 (44.5%)		
Time from first biopsy to actionable NGS results (days)	21 (15, 29)	19 (14, 25)	24 (18, 34)
Missing	20	4	16
Time from first biopsy to initiation of treatment (days)	35 (24, 49)	30 (22, 46)	37 (28, 49)
Missing	43	14	29

^1^ Median (IQR); n (%).

**Table 2:** Time from biopsy to NGS results and treatment initiation between NMH and CDH + Delnor.

Variable	CDH + Delnor, n = 106 ^1^	NMH, n = 85 ^1^	*p*-Value ^2^
Time from first biopsy to actionable NGS results	24 (18, 34)	19 (14, 25)	<0.001
Time from first biopsy to initiation of treatment	37 (28, 49)	30 (22, 46)	0.092

^1^ Median (IQR); ^2^ Wilcoxon rank sum test.

**Table 3:** Time from biopsy to NGS results and treatment initiation between comprehensive and non-comprehensive testing at CDH + Delnor.

Variable	Comprehensive, N = 29 ^1^	Non-Comprehensive, N = 61 ^1^	*p*-Value ^2^
Time from first biopsy to actionable NGS results	26 (21, 35)	22 (15, 33)	0.033
Time from first biopsy to initiation of treatment	37 (28, 49)	38 (29, 50)	0.6

^1^ Median (IQR); ^2^ Wilcoxon rank sum test.

## Data Availability

The original contributions presented in the study are included in the article or Supplementary Materials. Further inquiries can be directed to the corresponding author.

## Supplementary Materials

The following supporting information can be downloaded at: https://www.mdpi.com/article/10.3390/cancers16213654/s1, Figure S1: Median survival overall. Table S1: First-line targetable mutations seen between hospitals.
