# Supplementary material for: The Impact of Next-Generation Sequencing Workflows on Outcomes in Advanced Lung Cancer: A Retrospective Analysis at One Academic Health System
Source: Cancers (Basel). 2024 Oct 30;16(21):3654. doi: 10.3390/cancers16213654 (PMC11545180; doi:10.3390/cancers16213654)
Supplement: Supplementary file 1 [file cancers-16-03654-s001.zip › cancers-3233595-supplementary.pdf]

Supplementary Table 1: First-line targetable mutations seen between hospitals.

**NMH (n = 24)**	**CDH + Delnor (n = 19)**
EGFR L858R (7)<br>EGFR deletion 19 (5)<br>MET (4)<br>ALK (3)<br>EGFR – other (2)<br>ROS (1)<br>NTRK3 (1)<br>BRAF V600E (1)	EGFR deletion 19 (11)<br>EGFR L858R (4)<br>ALK (2)<br>EGFR – other (1)<br>BRAF V600E (1)<br>MET (1)

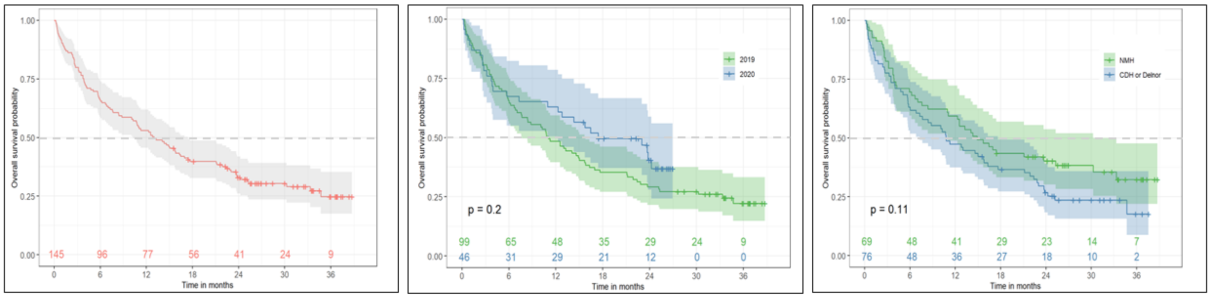

Supplementary Figure 1.
Figure S1a (left) shows overall survival across the populations, with median 13 months.
Figure S1b (center) shows overall survival in 2019 and 2020. Median OS in 2019 was 11.03 months and 17.43 months in 2020 (p = 0.2)
Figure S1c (right) shows overall survival by hospital site. Median OS at NMH was 14.9 months and 10.78 months at CDH (p = 0.11)
